# Research on remote sensing ecological livability index based on Google Earth Engine: a case study from Urumqi-Changji-Shihezi urban cluster

**DOI:** 10.7717/peerj.17872

**Published:** 2024-08-30

**Authors:** Mianwei Chen, Tianxing Wang, Yunqing Liu, Shikai Zhang, Yue Zhang

**Affiliations:** 1 School of Resources and Environment, Yili Normal University, Yining, Xinjiang, China; 2 Key Laboratory of Pollutant Chemistry and Environmental Treatment, Yili Normal University, Yining, Xinjiang, China

**Keywords:** Urumqi-Changji-Shihezi urban cluster, Google Earth Engine, RSELI, Remote sensing technology

## Abstract

The U-Chang-Shi (Urumqi-Changji-Shihezi) urban cluster, located at the heart of Xinjiang, boasts abundant natural resources. Over the past two decades, rapid urbanization, industrialization, and climate change have significantly threatened the region’s ecological livability. To comprehensively, scientifically, and objectively assess the ecological livability of this area, this study leverages the Google Earth Engine (GEE) platform and multi-source remote sensing data to develop a comprehensive evaluation metric: the Remote Sensing Ecological Livability Index (RSELI). This aims to examine the changes in the ecological livability of the U-Chang-Shi urban cluster from 2000 to 2020. The findings show that despite some annual improvements, the overall trend in ecological livability is declining, indicating that the swift pace of urbanization and industrialization has placed considerable pressure on the region’s ecological environment. Land use changes, driven by urban expansion and the growth in agricultural and industrial lands, have progressively encroached upon existing green spaces and water bodies, further deteriorating the ecological environment. Additionally, the region’s topographical features have influenced its ecological livability; large terrain fluctuations have made soil erosion and geological disasters common. Despite the central plains’ vast rivers providing ample water resources, over exploitation and ill-conceived hydrological constructions have led to escalating water scarcity. The area near the Gurbantunggut Desert in the north, with its extremely fragile ecological environment, has long been unsuitable for habitation. This study provides a crucial scientific basis for the future development of the U-Chang-Shi urban cluster and hopes to offer theoretical support and practical guidance for the sustainable development and ecological improvement of the region.

## Introduction

As the process of global urbanization continues to deepen, cities have become the main places where most people live and work. This rapid trend of urbanization, especially in developing countries, has brought a series of challenges and opportunities (Zhang et al., 2023). However, with the swift expansion of cities and population agglomeration, urban ecological environmental issues have gradually become a focal concern for both the public and governments. From air pollution and water scarcity to urban heat island effects, these environmental issues directly threaten the health and quality of life of urban residents and also constrain the sustainable development of cities (Chen et al., 2023). Therefore, the livability of cities, especially from an ecological perspective, has received high attention from governments, research institutions, and urban planners worldwide. Ecological livability involves not only the quality of the natural environment but also socioeconomic and cultural factors, making it an important indicator of a city’s overall development level (Li et al., 2020). In recent years, with the advancement of remote sensing technology, researchers have been able to obtain and analyze urban ecological environment data more accurately, thereby providing a scientific basis for assessing urban ecological livability.

**Figure 1:**
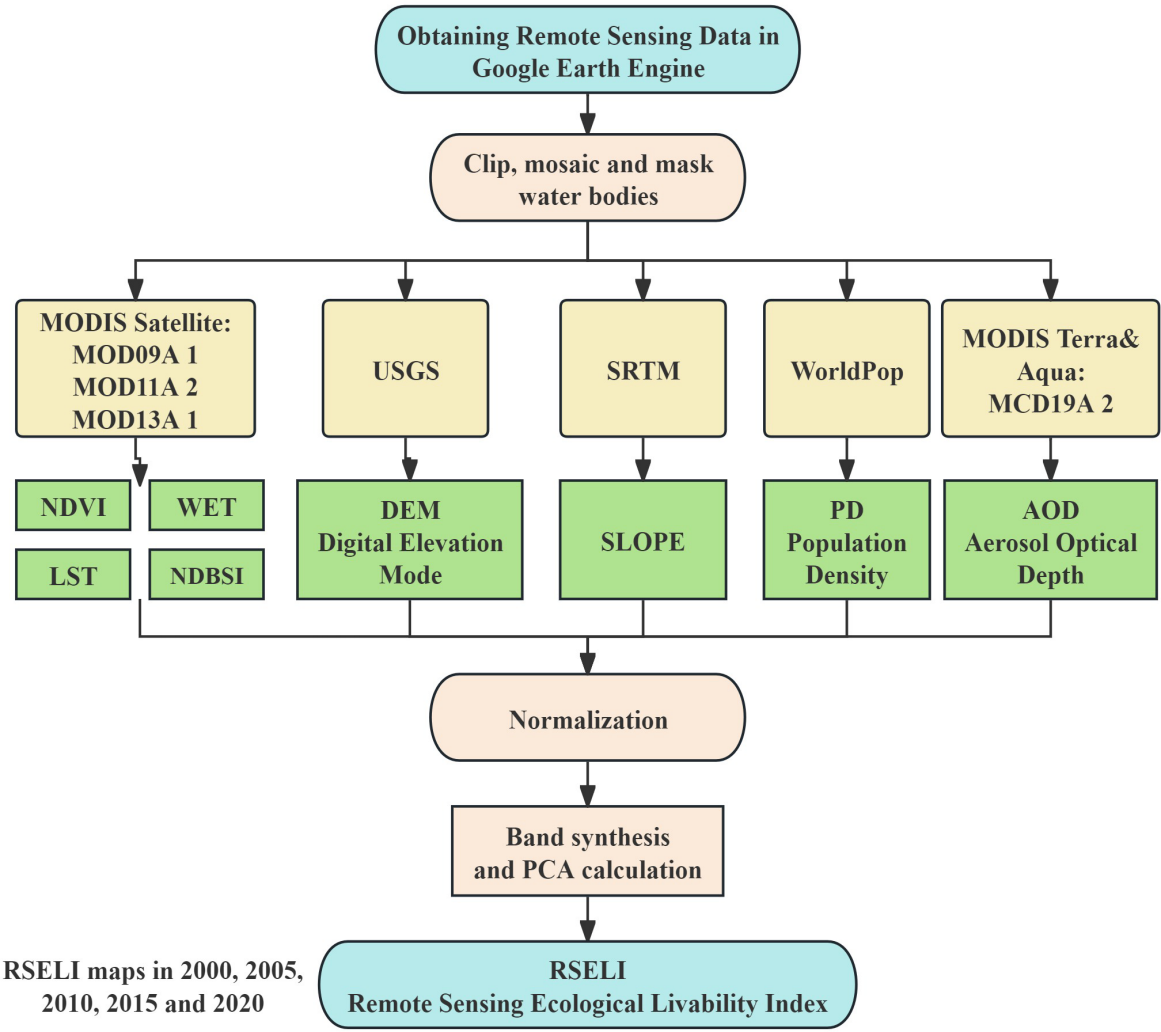
Workflow of the study.

The U-Chang-Shi urban cluster, a typical developing urban agglomeration in arid regions, faces widespread and representative challenges regarding ecological livability in the urbanization process. This area is characterized by arid climate, water scarcity, and fragile ecological environment, while also bearing the environmental pressures brought by rapid urbanization (Wang et al., 2024). The significant contradictions between urban development and ecological protection in this region necessitate more scientific, objective, and comprehensive evaluation methods to support urban ecological construction and management. Building on the research of previous scholars and considering the topographical and climatic characteristics of the U-Chang-Shi urban cluster, this study utilized the Google Earth Engine (GEE) platform to acquire eight key parameters: Greenness (NDVI), Wetness (WET), Dryness (NDBSI), Heat (LST), Elevation (DEM), Slope (SLOPE), Aerosol Optical Depth (AOD), and Population Density (PD). Workflow of the study is shown in Fig. 1. These parameters encompass multiple crucial aspects of the urban ecological environment and are key factors affecting urban livability. This research pioneers the application of principal component analysis to these eight parameters on the GEE platform, determining their weights to effectively avoid the biases of subjective weighting, and successfully constructing the Remote Sensing Ecological Livability Index (RSELI), which underscores the impact of the ecological environment on urban livability. Compared to traditional evaluation methods, the use of the GEE platform’s data acquisition and analysis capabilities has significantly enhanced the efficiency and accuracy of the study. This research not only provides a new method and perspective for assessing the ecological livability of the U-Chang-Shi urban cluster but also offers robust theoretical support and practical guidance for evaluating the ecological livability of other cities and urban clusters in typical dryland areas.

## Literature Review

To more precisely and systematically assess the ecological of cities, remote sensing (RS) and Geographic Information Systems (GIS) have played a crucial role in this field. Representing modern technology, their application in urban remote sensing has been widely recognized. Compared to traditional assessment methods, remote sensing technology can provide more extensive, real-time, and objective data, ensuring the scientific accuracy of evaluation results. Combined with GIS, researchers can conduct in-depth data analysis and visualization, thereby offering powerful decision support for policymakers. Berihun et al. (2019) used remote sensing data and GIS to analyze land use and cover changes from 1982 to 2017 in three watersheds of the Upper Blue Nile basin. The study found significant reductions in natural vegetation and increases in cultivated land, driven primarily by population growth and changes in agricultural practices. This research emphasizes the need for strategic land management in areas experiencing similar environmental changes (Berihun et al., 2019). In the study by Zhao et al. (2023) on the evaluation of the natural suitability of the human settlement environment on the northern slope of the Tianshan Mountains, GIS revealed the spatial distribution pattern of natural suitability for human settlement on the northern slope of the Tianshan Mountains. However, traditional GIS relies on manpower to process massive amounts of data, which can be overly complicated. Therefore, the Google Earth Engine (GEE) platform, as an advanced cloud computing remote sensing data platform, provides researchers worldwide with a vast array of satellite remote sensing data and powerful data processing tools (Zhang et al., 2023). Compared to traditional methods of obtaining and processing remote sensing data, the GEE platform offers comprehensive data, rapid processing, and easy operation advantages. Researchers no longer need to invest in expensive hardware or wait for lengthy data processing. The GEE platform can quickly complete complex computational tasks in the cloud, from simple image classification to complex model simulations (Zurqani et al., 2017). Moreover, GEE’s open Application Programming Interface (API) and flexible scripting language allow researchers to customize data processing and analysis according to their needs. This high degree of customizability enables the GEE platform to meet various complex research needs, from basic data visualization to advanced machine learning models, all easily implemented on the GEE platform (Liu et al., 2018). Xiong et al. (2017) developed the Automated Cropland Mapping Algorithm (ACMA) using GEE, employing satellite data to map Africa’s farmlands with an accuracy of about 90%, significantly reducing the time needed for agricultural surveys. Using GEE and Sentinel 5P imagery, a significant decrease in air pollution in the Ahvaz area of Iran was observed, with noted reductions in the concentrations of NO2 (13.7%), CO (6.1%), SO2 (28%), and HCHO (9.5%) before and after the COVID-19 pandemic. This highlights the platform’s versatility in meeting complex research needs, ranging from data visualization to machine learning applications (Fatemeh, Akbar & Khedri, 2023).

Starting from the basic components, structure, and behavior of the system, an ecological model can mathematically simulate the structure of the ecosystem with good physical significance, such as the pressure-state-response (PSR) model (Bai & Tang, 2010), ecological footprint model (Gu et al., 2015), urban resources and environment carrying capacity (URECC) system (Zhang et al., 2018), and ecosystem service assessment model (Zhang, Yang & Yu, 2006). However, some defects (*e.g.*, complicated process, and over-parameterization) bring considerable limitations to wider application. Using remote sensing data such as PM concentration, surface temperature, and vegetation coverage, a Comprehensive Evaluation Index (CEI) was established to assess environmental changes in many Chinese cities (He et al., 2017). In 2019, based on the analytic hierarchy process and expert scoring method, the Ecological Carrying Capacity (ECC) index was constructed according to the specific ecological conditions of the Aral Sea region (Wu et al., 2020). Additionally, the Urban Ecological Quality Index (UEQI) (Gu et al., 2015), the Ecological Footprint Variation Index (EFVI) (Musse, Barona & Rodriguez, 2018), the Potential Ecological Risk Index (Xu et al., 2008), and the City Sustainability Index (CSI) (Mori & Christodoulou, 2012) have also been widely applied.

In 2013, Xu et al. (2018) utilized Landsat satellite data to calculate four environmental factors—NDVI, WET, NDBSI, and LST—and employed Principal Component Analysis (PCA) to derive the Remote Sensing Ecological Index (RSEI) for evaluating the ecological environment of Fuzhou, which gained wide recognition globally. For instance, Alwan & Aziz (2022) used RSEI to assess ecological changes in Iraq’s Al-Hawizeh Marsh from 1990 to 2020; Wu et al. (2022) combined GEE and RSEI to analyze the ecological quality of the Sahel region in Africa from 2001 to 2020; Huang et al. (2024) constructed a seasonal RSEI using MODIS satellite data to evaluate the seasonal changes in ecological quality in the Jing-Jin-Ji area from 2001 to 2020. However, models solely based on natural environmental factors were insufficient for accurate ecological livability assessments. Consequently, some scholars have added additional factors to enhance evaluation models. For example, Yu et al. (2024) introduced the Ecological Livability Index (ELI) in 2022, incorporating NDVI, WET, NDBSI, LST, and AOD, weighted based on an entropy method, to assess the ecological livability of Wuhan across different seasons from 2002 to 2017. In 2023, Zhang et al. (2022) built upon the ELI to create the UELI (IMP) model, employing the harmonic analysis of time series (HANTS) and spatial–temporal information fusion based on a non-local means filter.

The application of the Remote Sensing Ecological Livability Index (RSELI) model offers several advantages and significance. Firstly, it leverages the vast data resources and processing capabilities of the Google Earth Engine (GEE) platform, allowing for real-time, extensive, and precise data analysis. Secondly, the use of principal component analysis ensures an objective weighting of key ecological parameters, minimizing subjective biases. This methodology enhances the accuracy and reliability of ecological livability assessments. The RSELI model not only provides a new perspective for evaluating the U-Chang-Shi urban cluster but also offers a robust framework for other cities and urban clusters, particularly in arid regions, supporting sustainable urban development and informed policy-making. This approach significantly improves the efficiency and effectiveness of ecological assessments, contributing valuable insights for urban planning and environmental management.

## Materials & Methods

### Research Area Overview

**Figure 2:**
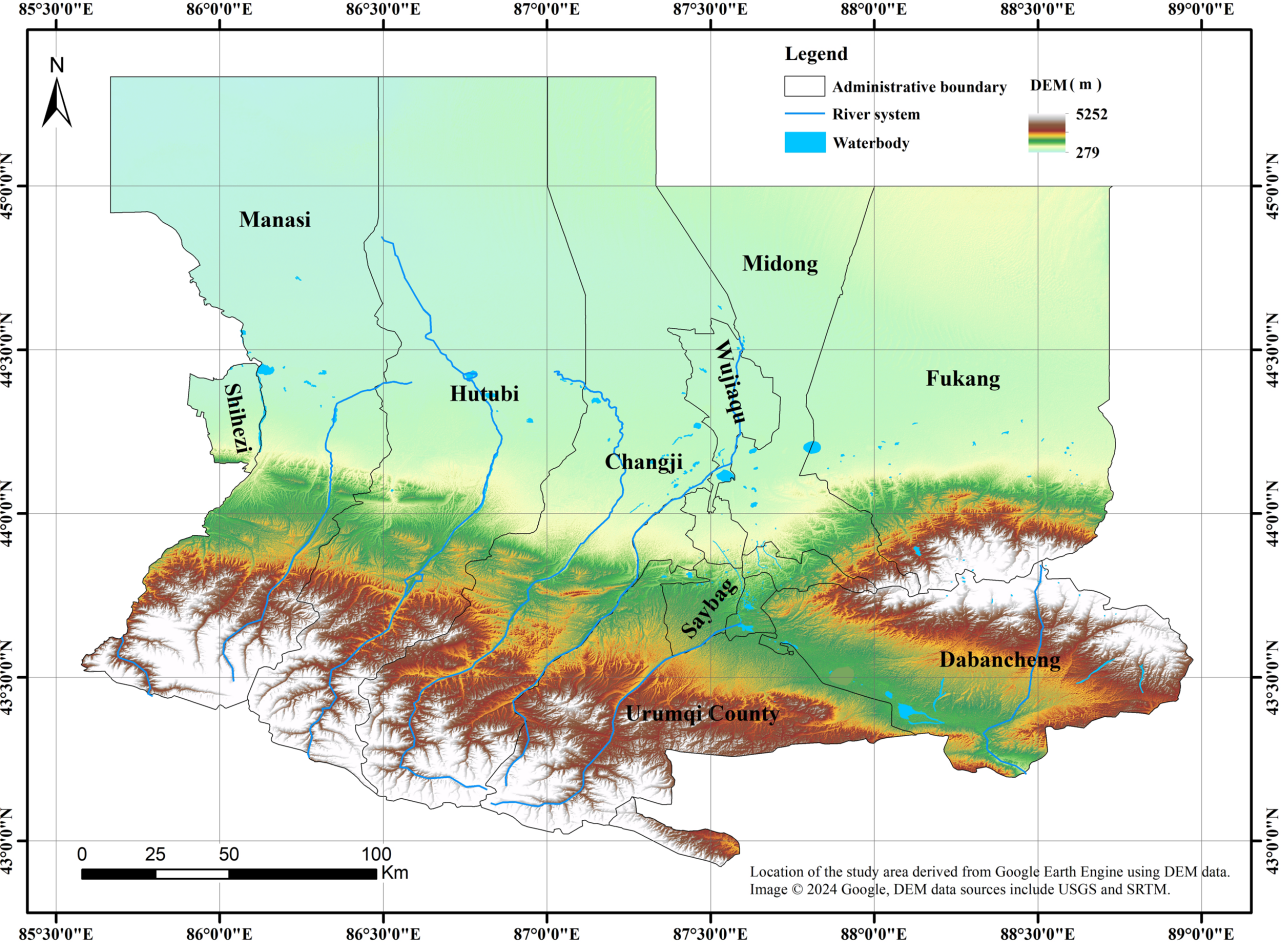
Schematic diagram of study area. The data used in the study were all obtained and processed through the Google Earth Engine (GEE) platform.

The U-Chang-Shi urban cluster is one of the 19 important city clusters planned by China and is the center of future development in Xinjiang, as well as its lifeline. It mainly includes Urumqi City, parts of Changji Hui Autonomous Prefecture, Wujiaqu City, and Shihezi City, with Changji, Shihezi, and Turpan serving as the regional center cities for urban cluster construction to drive the development of surrounding cities and to create a new western center centered on Urumqi. It is an important part of the economic belt on the northern slope of the Tianshan Mountains in Xinjiang (Qu, 2003). The research area is shown in Fig. 2.

The total area is 46,400 km^2^ (accounting for 2.8% of Xinjiang’s total area), and as of the end of 2021, the total permanent population was 5.717 million people (accounting for 30.2% of Xinjiang’s total), with a gross regional product of 537.182 billion yuan (accounting for 22.1% of Xinjiang’s total GDP). The region has a temperate continental arid climate, with an average annual temperature of 25.7 °C, an average annual rainfall of 275.5 mm, and is rich in ecological resources, with a wide variety of flora and fauna (Muhadaisi et al., 2021).

### Data sources and research methods

#### Data sources and preprocessing

The data used in the study were all obtained from the GEE platform, processed through GEE’s JavaScript code to compile data for the years 2000, 2005, 2010, 2015, and 2020. The four sets of data - greenness NDVI, wetness WET, dryness NDBSI, and heat LST - are derived from the American MODIS satellite data (Zhang, Zhou & Song, 2020). Elevation DEM and slope SLOPE data are from the United States Geological Survey (USGS) and the Shuttle Radar Topography Mission (SRTM) (Dong et al., 2022). Population density data are from the WorldPop project, which combines satellite and census data to generate high-resolution population maps (Dong et al., 2022; Huang et al., 2002) and AOD data for the region within China is sourced from MCD19A2 AOD data (Zhou et al., 2018).

The remotely sensed data utilized in this study were sourced from the Google Earth Engine (GEE) platform. To ensure the accuracy and consistency of the data, the following preprocessing steps were implemented. The cloud masking function within GEE was employed to identify and remove cloud cover through specific threshold settings and algorithms, thus obtaining clearer surface data. Cloud layers in MODIS satellite data were detected and masked using the Quality Assessment Band (QA Band). To ensure spatial alignment across images from different times and sensors, all imagery underwent geometric correction. While images in GEE are pre-corrected, additional ground control points were used as needed to ensure further alignment precision.Radiometric corrections were applied to ensure effective comparison across data from different times and satellites, using GEE’s standardized correction algorithms to eliminate radiometric discrepancies between images. Atmospheric corrections were conducted to mitigate the impacts of atmospheric scattering and absorption on image quality, with MODIS satellites employing the surface reflectance products in MOD09A1 for correction.Using boundary information from the study area, remotely sensed images were cropped to ensure consistency with the geographical scope of the study area, thereby minimizing interference from irrelevant regions. These data preprocessing operations were compiled, processed, and calculated using JavaScript code on the GEE platform, ensuring the efficiency and accuracy of constructing the Remote Sensing Ecological Livability Index (RSELI) model.

#### Remote sensing ecological livability index (RSELI)

In this study, the Remote Sensing Ecological Livability Index (RSELI) is derived using principal component analysis. It initially calculates the greenness (NDVI), wetness (WET), dryness (NDBSI), heat (LST), elevation (DEM), slope (SLOPE), Aerosol Optical Depth (AOD), and population density (PD) separately through models. To minimize errors, data from these eight indicators are selected within the 5% to 95% confidence interval and then normalized. The normalized data are combined through band synthesis, with the dataset of the eight normalized indicators concentrated on the first principal component. The weights are determined based on the contribution rates of the eight indicators on the principal component and their inherent characteristics, reducing the potential for human bias in assigning values. All these operations are executed using GEE’s JavaScript code for compilation and computation. The models for each indicator are as follows.

##### Greenness (NDVI).

NDVI has become the most widely applied vegetation index in remote sensing, used for monitoring surface green vegetation cover (Rouse et al., 1974). In this paper, this index is utilized to represent the greenness within the RSELI. The formula is as follows:

(1) \begin{eqnarray*}\text{NDVI}= \left( {\rho }_{\mathrm{NIR}}-{\rho }_{\mathrm{red}} \right) / \left( {\rho }_{\mathrm{NIR}}+{\rho }_{\mathrm{red}} \right) .\end{eqnarray*}

In the formula: *ρ*_NIR_ represents the near-infrared band; *ρ*_red_ represents the red band.

##### Wetness (WET).

WET can be calculated through the Tasseled Cap Transformation (K-T Transformation) (Crist et al., 1985; Baig et al., 2014), extracted from MODIS satellite imagery data. In this paper, this index is utilized to represent wetness within the RSELI. The formula is as follows:

(2) \begin{eqnarray*}\mathrm{WET}={\rho }_{\text{blue}}\times 0.0315+{\rho }_{\text{green}}\times 0.2021+{\rho }_{\mathrm{red}}\times 0.3102+{\rho }_{\mathrm{NIR}}\times 0.1594-\nonumber\\\displaystyle {\rho }_{\text{SWIR}1}\times 0.6806-{\rho }_{\text{SWIR}2}\times 0.6109.\end{eqnarray*}

In the formula: *ρ*_blue_ represents the blue band; *ρ*_green_ represents the green band; *ρ*_SWIR1_ represents short-wave infrared 1; *ρ*_SWIR2_ represents short-wave infrared 2.

##### Dryness (NDBSI).

NDBSI is effective for monitoring environmental dryness, calculated as the average of the Built-up Index (IBI) and the Bare Soil Index (SI) (Xu, 2008; Essa et al., 2012). In this paper, this index is used to represent dryness within the RSELI. The formula is as follows:

(3) \begin{eqnarray*}\mathrm{IBI}= \frac{ \frac{2{\rho }_{\text{SWIR}1}}{{\rho }_{\text{SWIR}1}+{\rho }_{\mathrm{NIR}}} - \frac{{\rho }_{\mathrm{NIR}}}{{\rho }_{\mathrm{NIR}}+{\rho }_{\mathrm{red}}} - \frac{{\rho }_{\text{green}}}{{\rho }_{\text{green}}+{\rho }_{\text{SWIR}1}} }{ \frac{2{\rho }_{\text{SWIR}1}}{{\rho }_{\text{SWIR}1}+{\rho }_{\mathrm{NIR}}} + \frac{{\rho }_{\mathrm{NIR}}}{{\rho }_{\mathrm{NIR}}+{\rho }_{\mathrm{red}}} + \frac{{\rho }_{\text{green}}}{{\rho }_{\text{green}}+{\rho }_{\text{SWIR}1}} } \end{eqnarray*}

(4) \begin{eqnarray*}\mathrm{SI}= \frac{ \left( {\rho }_{\text{SWIR}1}+{\rho }_{\mathrm{red}} \right) - \left( {\rho }_{\mathrm{NIR}}+{\rho }_{\text{blue}} \right) }{ \left( {\rho }_{\text{SWIR}1}+{\rho }_{\mathrm{red}} \right) + \left( {\rho }_{\mathrm{NIR}}+{\rho }_{\text{blue}} \right) } \end{eqnarray*}

(5) \begin{eqnarray*}\text{NDBSI}= \frac{\mathrm{IBI}+\mathrm{SI}}{2} \end{eqnarray*}

##### Heat (LST).

Heat (LST) involves the inversion of land surface temperature from satellite imagery using an atmospheric correction method. The emissivity (REf) of the surface is calculated through the Fractional Vegetation Cover (FVC). The original blackbody radiance values B(Ts), after radiometric correction, provide the radiance at the satellite, which is then atmospherically corrected to remove the effects of water vapor. Finally, the blackbody radiance is converted into land surface temperature using the Planck function (Jimenez-Munoz et al., 2009; Paolini, 2004; Weng, 2009; Nichol, 2005). The formula is as follows:

(6) \begin{eqnarray*}\mathrm{FV C}={\text{NDVI}}_{(< 0.05)}\times 0+{\text{NDVI}}_{(> 0.7)}\times 1+{\text{NDVI}}_{(0.05\sim 0.7)}\times \frac{{\text{NDVI}}_{(0.05\sim 0.7)}-0.05}{0.7-0.05} \end{eqnarray*}

(7) \begin{eqnarray*}{E}_{(\text{water})}=0.995\nonumber\\\displaystyle {E}_{(\text{building})}=0.9589+0.086\times \mathrm{FV C}-0.0671\times {\mathrm{FV C}}^{2}\nonumber\\\displaystyle {E}_{(\text{natural})}=0.9625+0.0614\times \mathrm{FV C}-0.0461\times {\mathrm{FV C}}^{2}\end{eqnarray*}

(8) \begin{eqnarray*}\mathrm{REf}={\text{NDVI}}_{(\leq 0)}\times {E}_{(\text{water})}+{\text{NDVI}}_{(0\sim 0.7)}\times {E}_{(\text{building})}+{\text{NDVI}}_{(\geq 0.7)}\times {E}_{(\text{natural})}\end{eqnarray*}

(9) \begin{eqnarray*}\mathrm{B} \left( \mathrm{Ts} \right) = \frac{{\rho }_{\text{TIRS}1}-{L}_{\mathrm{up}}-t\times (1-\mathrm{REf})\times {L}_{\text{down}}}{t\times \mathrm{REf}} \end{eqnarray*}

(10) \begin{eqnarray*}\mathrm{LST}= \frac{{\mathrm{K}}_{2}}{\ln \nolimits ( \frac{{\mathrm{K}}_{1}}{\mathrm{B} \left( \mathrm{Ts} \right) } +1)} .\end{eqnarray*}

In the formula: *E*_(water)_ represents the emissivity of water body pixels; *E*_(building)_ represents the emissivity of urban pixels; *E*_(natural)_ represents the emissivity of natural surface pixels; *L*_up_ represents the atmospheric upward radiance, *L*_down_ represents the atmospheric downward radiance, *t* represents the atmospheric transmittance in the thermal infrared band, and K_1_ and K_2_ are preset constants for satellite emission. All the above data are automatically acquired from the GEE platform.

##### Elevation (DEM) and slope (SLOPE).

DEM and SLOPE data used in GEE are from the USGS/SRTMGL1_003 dataset, jointly measured by the National Aeronautics and Space Administration (NASA) and the National Imagery and Mapping Agency (NIMA) of China. The data were collected using the Shuttle Radar Topography Mission (SRTM) system aboard the Endeavour space shuttle, leading to the creation of digital terrain models, which are the current SRTM terrain product data (Aizizi et al., 2023).

##### Aerosol optical depth (AOD).

AOD is selected to represent air quality conditions, reflecting the total amount of particulate matter suspended in the atmosphere. The MCD19A2 AOD data within the China region are chosen, which are the version 6 data products of the MODIS Terra and Aqua combined, using the Multi-Angle Implementation of Atmospheric Correction (MAIAC) land aerosol optical thickness (AOD) at grid level 2. These can be directly accessed and processed for annual average data compilation using JavaScript code on the GEE platform (Duan et al., 2021).

##### Population density (PD).

The WorldPop project employs machine learning methods to analyze the relationship between population density and a series of geospatial covariate layers, disaggregating the most recent census-based population counts matched to corresponding administrative units into approximately 100x100m grid cells. The mapping method is based on a random forest algorithm for dasymetric redistribution (Lloyd, Sorichetta & Tatem, 2017), allowing for direct extraction and utilization through the GEE platform.

##### RSELI model.

Due to the uneven dimensions of the eight factors, directly applying them in PCA would result in unbalanced indicator weights. Therefore, it is necessary to normalize these eight factors before performing PCA, converting each indicator value into a dimensionless value within the range of 0 to 1. This normalization ensures that all factors are on a comparable scale, allowing for a more accurate and balanced PCA calculation (Amani et al., 2020). The formula for this normalization process is as follows:

(11) \begin{eqnarray*}{\text{XI}}_{\text{i}}= \frac{{\mathrm{I}}_{\mathrm{i}}-{\text{I}}_{\mathrm{min}}}{{\text{I}}_{\mathrm{max}}-{\text{I}}_{\mathrm{min}}} .\end{eqnarray*}

In the formula: XI_i_ represents the value after normalization, I_i_ represents the value before normalization, and I_max_ and I_min_ represent the maximum and minimum values before normalization, respectively.

After normalization of the eight factors, the first principal component (PC1) is calculated using the band synthesis and principal component analysis modules within the GEE platform. The formula for calculating PC1 is as follows:

(12) \begin{eqnarray*}\text{RSELI}=\mathrm{PC}1 \left[ \text{NDVI},\mathrm{WET},\mathrm{DEM},\text{SLOPE},\mathrm{AOD},\text{NDBSI},\mathrm{LST},\mathrm{PD} \right] .\end{eqnarray*}

The model described above can be compiled and processed using JavaScript code in the Google Earth Engine (GEE). GEE allows for the direct provision of the final results in TIFF format, which can be downloaded for analysis and use. Leveraging the GEE platform significantly reduces processing time due to its cloud computing capabilities, enabling the handling of multi-year remote sensing imagery in one go. This efficiency is particularly beneficial for extensive datasets covering long time periods, making GEE a powerful tool for environmental and ecological research.

## Results

### Analysis of PCA

The PCA’s results for the eight factor indicators of the U-Chang-Shi urban cluster from 2000 to 2020, as shown in Table 1, reveal that the first principal component (PC1) accounted for contribution rates of 78.59%, 76.57%, 77.32%, 78.23%, and 77.45% for the five years, respectively. All contribution rates exceeded 75%, indicating that PC1 encompasses the vast majority of the characteristics among the eight indicators and can effectively represent the ecological livability level of the U-Chang-Shi urban cluster. The positive values of greenness and wetness across all five years in the table suggest that the region’s vegetation cover and moisture level positively impact its ecological livability. In contrast, other indicators negatively affect the ecological condition, which aligns with the expected ecological status due to the arid continental climate prevalent in the area.

**Table 1:** Principal component analysis of indicators from 2000 to 2020.

Years	Eigenvalues	PC 1	PC 2	PC 3	PC 4	PC 5	PC 6	PC 7	PC 8
2000	NDVI	0.3974	0.8290	0.1511	0.1779	0.0181	−0.3162	−0.0090	−0.0017
	WET	0.1127	0.0231	−0.1449	−0.8986	−0.0549	−0.3855	−0.0942	0.0022
	DEM	−0.6121	0.4106	0.3067	−0.1983	−0.3785	0.3280	−0.2692	0.0024
	SLOPE	−0.4178	0.3032	−0.7940	0.0903	0.3036	−0.0401	−0.0345	0.0012
	AOD	−0.0991	0.0755	0.3832	−0.2016	0.8693	0.1947	−0.0594	−0.0005
	NDBSI	−0.1029	−0.2048	0.0894	0.2461	0.0706	−0.4579	−0.8150	−0.0044
	LST	−0.5117	−0.0637	0.2773	0.1107	0.0254	−0.6282	0.4996	−0.0032
	PD	−0.0003	0.0011	−0.0022	−0.0040	−0.0015	0.0044	0.0011	−1.0000
	Contribution rate/%	78.59%	11.32%	4.17%	3.07%	1.32%	0.76%	0.43%	0.34%
2005	NDVI	0.3207	0.7897	0.3442	−0.1524	−0.3269	−0.0880	0.1314	−0.0017
	WET	0.2062	0.0388	−0.6243	0.5065	−0.4808	−0.2530	0.1206	0.0054
	DEM	−0.6026	0.3848	0.0949	0.4672	0.3530	−0.3592	0.0891	0.0021
	SLOPE	−0.4128	0.2707	−0.5837	−0.6412	−0.0435	−0.0411	0.0301	0.0015
	AOD	−0.0575	0.0615	0.0432	0.0065	−0.1840	−0.2342	−0.9499	−0.0003
	NDBSI	−0.1315	−0.3815	0.2989	−0.2695	−0.3224	−0.7184	0.2347	−0.0034
	LST	−0.5482	−0.0651	0.2255	0.1345	−0.6306	0.4760	0.0450	−0.0012
	PD	−0.0002	0.0014	−0.0059	0.0037	0.0006	−0.0001	0.0001	−1.0000
	Contribution rate/%	76.57%	12.38%	4.21%	3.40%	1.45%	0.85%	0.76%	0.38%
2010	NDVI	0.3258	−0.8280	−0.1107	0.3324	−0.1635	0.2101	0.1209	−0.0020
	WET	0.2102	−0.1017	0.0152	−0.7783	0.1560	0.5336	0.1745	0.0069
	DEM	−0.6421	−0.3794	−0.3881	−0.1785	0.3818	−0.2542	0.2256	0.0010
	SLOPE	−0.4439	−0.2574	0.8443	−0.0414	−0.1283	0.0719	0.0213	0.0015
	AOD	−0.1019	−0.0709	−0.1978	−0.3845	−0.8420	−0.2951	0.0388	−0.0031
	NDBSI	−0.0687	0.2744	−0.0005	0.2399	−0.1448	0.1773	0.9000	−0.0052
	LST	−0.4746	0.1162	−0.2914	0.2116	−0.2381	0.6949	−0.3034	0.0010
	PD	−0.0003	−0.0009	0.0015	−0.0061	0.0047	0.0038	−0.0039	−1.0000
	Contribution rate/%	77.32%	11.56%	4.08%	3.56%	1.40%	0.83%	0.75%	0.50%
2015	NDVI	0.3509	−0.8436	−0.2239	−0.2259	0.0060	0.2531	−0.0011	−0.0057
	WET	0.2250	−0.0514	0.4590	0.6302	0.3332	0.4774	0.0058	−0.0047
	DEM	−0.6167	−0.4000	−0.2230	0.3992	0.3777	−0.3285	0.0022	−0.0015
	SLOPE	−0.4257	−0.2760	0.7658	−0.3744	−0.1253	0.0164	0.0015	−0.0017
	AOD	−0.0896	−0.1711	−0.0142	0.4842	−0.8532	−0.0063	0.0007	−0.0009
	NDBSI	−0.0037	0.0070	−0.0034	−0.0027	−0.0014	0.0003	0.0043	−0.9999
	LST	−0.5067	0.1421	−0.3206	−0.1335	−0.0514	0.7745	−0.0016	0.0046
	PD	−0.0003	−0.0010	0.0041	0.0047	0.0021	0.0006	−1.0000	−0.0044
	Contribution rate /%	78.23%	11.68%	3.97%	3.13%	1.33%	0.80%	0.47%	0.39%
2020	NDVI	0.3570	−0.8079	−0.3083	−0.1195	0.0812	0.3200	0.0385	−0.0019
	WET	0.2278	−0.0904	0.7467	0.4665	0.1611	0.3578	0.1036	0.0055
	DEM	−0.5793	−0.3982	−0.0429	0.3805	0.3079	−0.3781	0.3485	0.0036
	SLOPE	−0.3967	−0.2661	0.5227	−0.6902	−0.1379	0.0481	0.0285	0.0016
	AOD	−0.1911	−0.1600	−0.0386	0.3324	−0.8995	0.1179	0.0543	−0.0059
	NDBSI	−0.0994	0.2847	−0.1954	−0.1419	0.0372	0.4846	0.7839	−0.0037
	LST	−0.5303	0.0556	−0.1804	0.1273	0.2079	0.6129	−0.4981	−0.0009
	PD	−0.0002	−0.0010	0.0065	0.0015	0.0066	−0.0029	−0.0010	−1.0000
	Contribution rate /%	77.45%	11.25%	4.35%	3.52%	1.37%	0.89%	0.71%	0.46%

Observing the absolute values of the eigenvalues of the eight indicators in Table 1 from 2000 to 2020, the ranking of the absolute values of the indicator eigenvalues for the top five positions consistently follows the order of DEM >LST >SLOPE >NDVI >WET. From 2000 to 2005, NDBSI >AOD, and from 2005 to 2020, AOD >NDBSI. In all years, PD consistently ranks last, indicating that the main factors affecting the ecological livability of the region are elevation, temperature, slope, greenness, and wetness, in that order. Given the region’s location on the northern slope of the mid-section of the Tianshan Mountains, elevation and slope notably limit its ecological livability, with temperature impact ranked second. This is attributable to the presence of a vast desert area in the northern part of the region (the Gurbantunggut Desert), which experiences significant diurnal temperature variations, thereby affecting greenness and wetness, in line with characteristics typical of arid areas. Dryness and Aerosol Optical Depth show that before 2005, dryness had a greater impact than Aerosol Optical Depth, but thereafter, the impact of Aerosol Optical Depth exceeded that of dryness, mainly due to rapid industrial development in the region and a surge in emissions of atmospheric pollutants. Huang’s study on the spatiotemporal variation of aerosol optical thickness in Xinjiang from 2000 to 2013 noted an annual increase in AOD in economically developed areas of Northern Xinjiang, especially in the economic belt of the northern slope of the Tianshan Mountains, including the Du-Kui-Wu area (Dushanzi-Kuitun-Wusu), Shihezi, and the U-Chang region, where AOD values showed an upward trend. This conclusion aligns with the findings of this study (Huang et al., 2015). As for the near-zero eigenvalue of population density, it indicates that population density has almost no impact on the ecological livability of the region.

### Spatio-temporal analysis of ecological livability levels in the U-Chang-Shi urban cluster

Based on most researchers’ studies on ecological livability, it is typically categorized into five levels. The RSELI index uses 0.2 as the benchmark for grading (Shi & Li, 2021; Fu & Zhang, 2023; Liu, Wu & Jin, 2023; Zhang et al., 2023), which are as follows: Excellent (Livable, 0.8∼1.0), Good (Relatively Livable, 0.6∼0.8), Moderate (Moderately Livable, 0.4∼0.6), Fair (Unlivable, 0.2∼0.4), and Poor (Extremely Unlivable, 0.0∼0.2).

Statistics from Table 2 indicate that the ecological livability index of the U-Chang-Shi urban cluster has slightly decreased over the past 20 years, with a significant decline between 2000 and 2005 and a gradual improvement thereafter, maintaining an overall state of Moderate livability. The AOD indicator has significantly increased over the 20 years, associated with air pollution, dust storms, and soil dust caused by drought. This period saw rapid industrial development in the area, leading to a significant increase in the emission of atmospheric pollutants. The overall change in LST has not been significant, showing a trend towards stability. The NDBSI indicator has slightly decreased over the 20 years, indicating a reduction in the region’s dryness level, benefiting from an overall increase in the NDVI indicator over the same period, which reflects increased vegetation volume primarily due to large-scale vegetation planting and windbreak and sand fixation projects in Xinjiang’s desert areas over the last 20 years, reducing soil erosion. Although the NDVI indicator has increased annually, the WET indicator has gradually decreased over 20 years, consistent with the region’s arid climate. Based on the analysis of precipitation change characteristics in Urumqi, there has been a yearly decrease in the number of rainy days over the past 30 years, with frequent occurrences of extreme heavy rainfall and drought events, leading to a reduction in humidity (Su & Xie, 2018).

**Table 2:** Statistical values of various factors and RSELI from 2000 to 2020.

Factor	Years				
	2000	2005	2010	2015	2020
RSELI	0.519	0.459	0.479	0.493	0.490
AOD	0.364	0.394	0.464	0.541	0.712
LST (°C)	41.407	41.301	40.359	41.130	40.530
NDBSI	0.112	0.109	0.097	0.108	0.104
NDVI	0.295	0.300	0.323	0.308	0.317
WET	−0.156	−0.203	−0.202	−0.209	−0.224
PD (People/m^2^)	24.900	28.605	34.068	38.989	45.060

**Notes.**

Except for Heat (LST) and Population Density (PD), the other factors are dimensionless.

**Figure 3:**
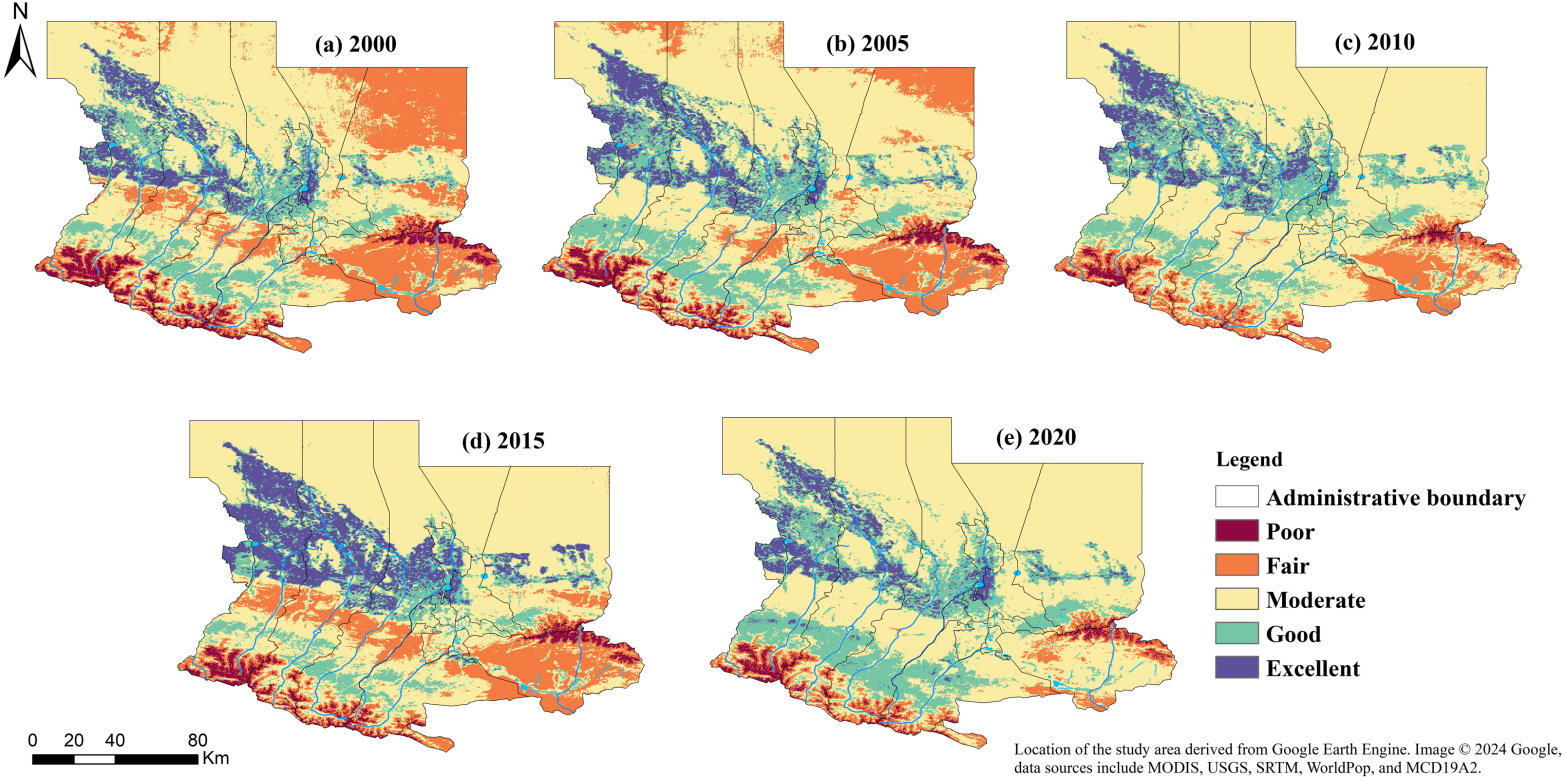
(A–E) Spatial distribution of RSELI in U-Chang-Shi Urban Cluster from 2000 to 2020. The data used in the study were all obtained and processed through the Google Earth Engine (GEE) platform.

Based on the aforementioned RSELI grading standards, the RSELI of the U-Chang-Shi urban cluster for each year was reclassified using ArcGIS 10.5 software, resulting in the Ecological Livability Map of the U-Chang-Shi urban cluster from 2000 to 2020, as shown in Fig. 3.

Combining the topographic map in Fig. 2 and the remote sensing ecological livability map in Fig. 3 reveals that the terrain in the U-Chang-Shi urban cluster distinctly shows a high south and low north characteristic. The southern region, especially the southwest and southeast areas close to the Tianshan Mountains, has significant topographical variations, which puts pressure on its ecological livability, even leading to potential soil erosion and geological disasters. In contrast, the central area is a vast plain. As can be seen from the map, the U-Chang-Shi urban cluster is home to numerous rivers, especially Changji, located between two main rivers, bringing abundant water resources to the area. This positively supports local agriculture and the ecological environment. The northern region, near the Gurbantunggut Desert, has an extremely fragile ecosystem and has been unsuitable for habitation over the past 20 years. The long-term pressure on the ecological environment in these fragile areas requires effective ecological restoration and protection.

Over the past 20 years, with the development of cities like Urumqi, Changji, and Shihezi, the trend of urban expansion has become increasingly apparent. Urbanization-induced land-use changes, reduction of green spaces, and increased pollution emissions may impact the local ecological environment. Since 2010, as indicated in Fig. 3, with increased attention to the ecological environment, the U-Chang-Shi urban cluster has been actively engaged in greening and constructing parks and other ecological projects. These measures have played a positive role in improving and protecting the local ecological environment and enhancing the ecological livability. Of course, for sustainable development, the region needs to consider factors such as topography, water resources, urbanization, and economic development comprehensively. Strengthening ecological protection and rational resource utilization, maintaining a balance between ecology and economic development, and avoiding ecological destruction caused by excessive development are crucial.

Using ArcGIS and Excel, the area of each RSELI level for five periods from 2000 to 2020 was extracted and analyzed, as shown in Table 3. Table 3 indicates that over 20 years, the area classified as Extremely Unlivable showed a trend of initial increase, followed by a decrease, and then another increase, mainly due to sand erosion caused by extreme weather events, resulting in fluctuations. Overall, the area under this category increased over 20 years. The area of the Unlivable level increased from 0.278 to 1.130 thousand km^2^ in 2005, nearly quadrupling, but then gradually decreased to 0.707 thousand km^2^ by 2020. Although it was an increase from 2000, it was significantly lower than the peak in 2005, indicating some improvement in ecological livability. The area of the Moderately Livable level decreased from 2.919 thousand km^2^ in 2000 to 2.529 thousand km^2^ in 2020. Despite still covering a large area, this reduction over 20 years should be noted as a warning against ecological degradation. The area of the Relatively Livable level fluctuated significantly over 20 years but overall showed a decreasing trend, with a reduction of 7.73%. The area classified as Livable doubled from 0.259 thousand km^2^ in 2000 to 0.520 thousand km^2^ in 2020, showing a clear growth trend. In summary, these data indicate that more areas have become more suitable for living. At the same time, the total areas classified as Unlivable and Extremely Unlivable have also increased, which may reflect environmental degradation or pressures from urbanization in some regions.

**Table 3:** Area and proportion of RSELI grades in the U-Chang-Shi Urban Cluster from 2000 to 2020.

Years	Poor		Fair		Moderate		Good		Excellent	
	Area/ 10^4^ km^2^	Proportion	Area/ 10^4^ km^2^	Proportion	Area/ 10^4^ km^2^	Proportion	Area/ 10^4^ km^2^	Proportion	Area/ 10^4^ km^2^	Proportion
2000	0.088	1.97%	0.278	6.23%	2.919	65.44%	0.917	20.57%	0.259	5.80%
2005	0.161	3.60%	1.130	25.34%	2.239	50.19%	0.665	14.91%	0.266	5.96%
2010	0.141	3.16%	0.796	17.84%	2.372	53.18%	0.828	18.56%	0.324	7.26%
2015	0.064	1.42%	0.472	10.58%	2.804	62.88%	0.845	18.95%	0.275	6.17%
2020	0.132	2.96%	0.707	15.84%	2.529	56.71%	0.572	12.84%	0.520	11.65%

### Spatio-temporal analysis of the variations in ecological livability in the U-Chang-Shi urban cluster

**Figure 4:**
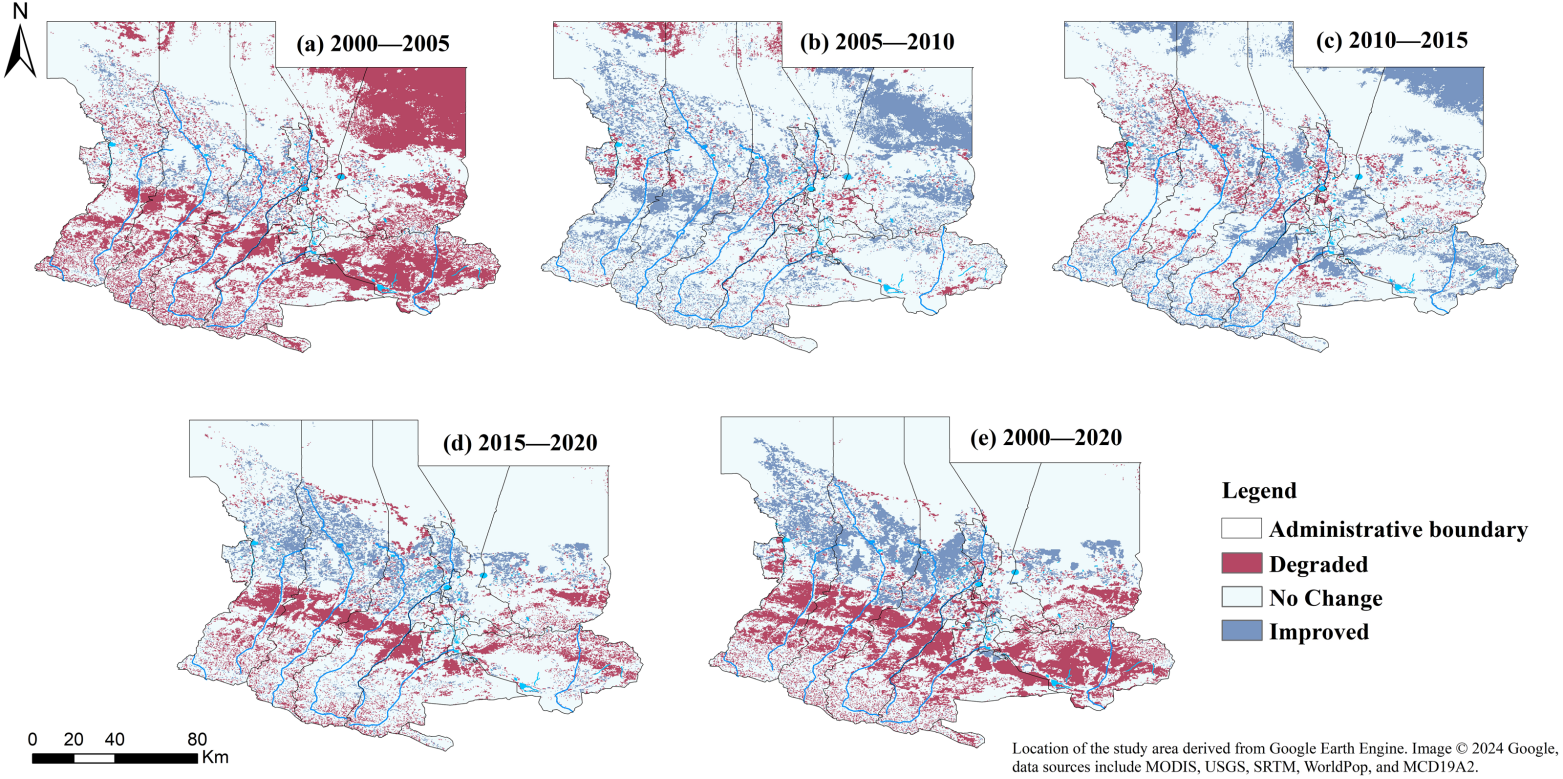
(A–E) Temporal and spatial distribution of RSELI in U-Chang-Shi Urban Cluster from 2000 to 2020. The data used in the study were all obtained and processed through the Google Earth Engine (GEE) platform.

Based on the RSELI classification, to obtain spatio-temporal distribution information on the changes in ecological environment quality between different years, the raster calculator in ArcGIS was used to process RSELI of different years. The changes were categorized into three types: improved (>0), unchanged (=0), and worsened (<0), with the statistical results shown in Table 4. The results were visualized using ArcGIS 10.5, as seen in Fig. 4.

**Table 4:** Changes of ecological livability in the U-Chang-Shi Urban Cluster from 2000 to 2020.

Classification	2000–2005		2005–2010		2010–2015		2015–2020		2000–2020	
	Area/ 10^4^ km^2^	Proportion	Area/ 10^4^ km^2^	Proportion	Area/ 10^4^ km^2^	Proportion	Area/ 10^4^ km^2^	Proportion	Area/ 10^4^ km^2^	Proportion
Degraded	1.37	30.78%	0.22	4.93%	0.30	6.82%	0.56	12.60%	0.89	19.88%
No Change	2.95	66.19%	3.38	75.75%	3.46	77.69%	3.50	78.49%	3.11	69.79%
Improved	0.13	3.03%	0.86	19.33%	0.69	15.49%	0.40	8.91%	0.46	10.33%

From 2000 to 2020, the region’s livability experienced significant changes, with a large area showing worsened livability between 2000 and 2005, while from 2005 to 2010 and 2015 to 2020, more areas saw improvements in livability. Throughout 2000 to 2020, the areas with unchanged livability accounted for the largest proportion, averaging 69.79%, indicating that despite changes in some regions, the livability of most areas remained stable. The proportion of areas with worsened livability was highest between 2000 and 2005, reaching 30.78%, with this percentage gradually decreasing thereafter. Overall, the proportion of areas with worsened livability from 2000 to 2020 was 19.88%. Areas with improved livability had the highest proportion between 2005 and 2010, at 19.33%, with an average proportion of 10.33% throughout 2000 to 2020.

## Discussion

This study’s findings underscore the dynamic interplay between urbanization, industrialization, and the ecological livability of the U-Chang-Shi urban cluster. The use of the RSELI, derived from comprehensive remote sensing data *via* the Google Earth Engine (GEE), provides a detailed understanding of ecological livability trends over the past two decades. Despite improvements in ecological livability in certain years, the overall trend indicates a gradual decline in ecological livability, highlighting the significant impact of rapid urban and industrial expansion.

The analysis reveals a direct correlation between eight environmental factors and the degradation of ecological quality. This observation is particularly relevant given the region’s industrial boom and its consequent environmental repercussions. The study emphasizes the significant influence of geographical features on ecological livability. The disparity in livability between the southern mountainous regions and the central plains of the U-Chang-Shi urban cluster exemplifies how natural topography can exacerbate or alleviate the challenges posed by urbanization. The vulnerability of northern areas, close to the Gurbantunggut Desert, to becoming uninhabitable zones further underscores the necessity for strategic ecological management and conservation efforts.

The spatio-temporal analysis based on the RSELI classification elucidates the varying degrees of ecological livability across different areas within the urban cluster, offering a granular perspective on the distribution of livable spaces. This detailed mapping aids in identifying areas in dire need of ecological restoration and those showing resilience or improvement in livability standards, facilitating targeted intervention. In light of these findings, it becomes crucial for policymakers and urban planners to adopt an integrated approach to urban development that equally prioritizes ecological sustainability alongside economic and infrastructural expansion. The significant role of green spaces, water conservation, and pollution control measures in enhancing urban livability cannot be overstated. Moreover, the study’s methodology, leveraging the advanced capabilities of the GEE platform, sets a precedent for future ecological and urban research, providing a scalable and efficient framework for monitoring and analyzing global environmental quality.

In contrast to prior research, which predominantly emphasized human-centric indicators and employed survey methodologies for assessing ecological livability—illustrated by Zhu, who utilized local yearbook data combined with surveys, and Pan, who integrated remote sensing imagery with survey data—this study encompasses both ecological and anthropogenic factors. It incorporates eight distinct variables through the Google Earth Engine (GEE) platform, enabling precise and efficient computational analysis. This approach offers a significant advancement over models that solely relied on vegetation and landscape pattern indices without incorporating human dimensions, underscoring the comprehensive and timely nature of this research.

Unlike other studies, previous ecological suitability evaluations focused more on humanistic indicators and were conducted through survey questionnaires. For example, Zhu et al. (2020) conducted Community Level Livability evaluations using local yearbook data and survey questionnaires, while Pan et al. (2023) used remote sensing image data and survey questionnaires. However, this approach was inefficient and data updates were not timely. Other scholars have also constructed evaluation models based on six ecological indicators, namely vegetation conditions and landscape patterns (Huang, Li & Zhang, 2022), but lack humanistic indicators. Therefore, compared with other scholars’ research, this study selected eight factors, including ecological and humanistic categories. The entire calculation process was carried out through the GEE platform, achieving accurate and efficient results.

Although the U-Chang-Shi urban cluster faces considerable ecological challenges, the insights gained from this study offer a roadmap for sustainable urban development. By integrating ecological considerations into the urban planning process, it is possible to pave the way towards a more livable, resilient, and sustainable urban future.The findings of this study provide crucial insights for urban planners and policymakers in the region, aiding them in incorporating ecological conservation and sustainable development into their decision-making processes for future urban expansion. This research not only offers a reference model for assessing ecological livability in similar regions but also identifies directions for further investigation. Future developments could include more sophisticated ecological monitoring systems that integrate finer remote sensing data and other environmental variables to rapidly and comprehensively assess the ecological livability of regions, offering more precise data support for urban planners and policymakers. Additionally, by building on these monitoring systems and incorporating machine learning and big data analytics, future research can more effectively identify key drivers of changes in ecological livability, providing a scientific basis for devising differentiated ecological protection and restoration strategies. Considering regional geographic conditions, climate change, and socio-economic factors collectively will aid in achieving sustainable development goals for the U-Chang-Shi urban cluster and other similar areas, ensuring the coordinated development of ecology and economy.

## Conclusion

This study, based on the Google Earth Engine (GEE) platform and utilizing remote sensing data, constructed the RSELI index to conduct an in-depth analysis of the ecological livability of the U-Chang-Shi urban cluster from 2000 to 2020. The findings are as follows.

Spatio-temporal variation in ecological livability: The ecological livability index of the U-Chang-Shi urban cluster fluctuated slightly over 20 years but generally remained in a moderately livable state. Although there was a significant decline from 2000 to 2005, the index gradually began to rise from 2005, which is related to regional ecological restoration projects and greening activities.

Changes in key ecological factors: The AOD indicator significantly increased over 20 years, closely related to regional industrial development and increased emissions of atmospheric pollutants; the NDVI indicator showed an overall increase in vegetation in the area, a direct result of China’s large-scale vegetation planting and windbreak and sand fixation projects in Xinjiang’s desert areas over the last 20 years.

Geography and ecological livability: The topographical characteristics of the U-Chang-Shi urban cluster have significantly impacted its ecological livability. The southern region, especially parts bordering the Tianshan Mountains, due to large topographical variations, has relatively lower ecological livability, while the central plains, benefiting from abundant river resources, have a relatively better ecological environment.

Urbanization and ecological livability: The acceleration of urbanization, changes in land use, reduction of green spaces, and increase in pollution may adversely affect the ecological environment of the U-Chang-Shi urban cluster. However, since 2010, the region has shown a marked increase in attention to the ecological environment, with numerous greening and ecological restoration projects implemented, positively impacting ecological livability.

In summary, although the ecological livability of some areas has improved, many areas, especially the fragile ecological regions near the Gurbantunggut Desert in the north, are still experiencing a decline in ecological livability. The ecological restoration and protection tasks in these areas remain challenging. To achieve sustainable development, the U-Chang-Shi urban cluster should consider multiple factors such as topography, water resources, urbanization, and economic development comprehensively. It should balance the relationship between ecological and economic development, strengthen ecological protection, use resources rationally, and avoid ecological damage caused by excessive development.

## Additional Information and Declarations

### Data Availability

The following information was supplied regarding data availability: The original data is from Google Earth Engine (GEE) open data. 1. Normalized Difference Vegetation Index (NDVI): MOD13A2.061 Terra Vegetation Indices 16-Day Global 1km, https://developers.google.com/earth-engine/datasets/catalog/MODIS_061_MOD13A2
2. Wetness (WET): MYD09A1.061 Aqua Surface Reflectance 8-Day Global 500m, https://developers.google.com/earth-engine/datasets/catalog/MODIS_061_MYD09A1
3. Normalized Difference Built-up and Soil Index (NDBSI): USGS Landsat 8 Level 2, Collection 2, Tier 1, https://developers.google.com/earth-engine/datasets/catalog/LANDSAT_LC08_C02_T1_L2
4. Land Surface Temperature (LST): MOD11A2.061 Terra Land Surface Temperature and Emissivity 8-Day Global 1km, https://developers.google.com/earth-engine/datasets/catalog/MODIS_061_MOD11A2
5. Digital Elevation Model (DEM): NASA SRTM Digital Elevation 30m, https://developers.google.com/earth-engine/datasets/catalog/USGS_SRTMGL1_003
6. Slope (SLOPE): NASA SRTM Digital Elevation 30m, https://developers.google.com/earth-engine/datasets/catalog/USGS_SRTMGL1_003
7. Aerosol Optical Depth (AOD): MOD04_L2 - MODIS/Terra Aerosol 5-Min L2 Swath 10km, https://ladsweb.modaps.eosdis.nasa.gov/missions-and-measurements/products/MOD04_L2
8. Population Density (PD): GPWv411: UN-Adjusted Population Density (Gridded Population of the World Version 4.11), https://developers.google.com/earth-engine/datasets/catalog/CIESIN_GPWv411_GPW_UNWPP-Adjusted_Population_Density
